# Nitric oxide secretion in human conjunctival fibroblasts is inhibited by alpha linolenic acid

**DOI:** 10.1186/s12950-015-0104-1

**Published:** 2015-10-25

**Authors:** Nir Erdinest, Noam Shohat, Eli Moallem, Claudia Yahalom, Hadas Mechoulam, Irene Anteby, Haim Ovadia, Abraham Solomon

**Affiliations:** Departments of Ophthalmology, Hadassah-Hebrew University Medical Center, Jerusalem, Israel; Clinical Immunology and Allergy Unit, Hadassah-Hebrew University Medical Center, Jerusalem, Israel; Department of Neurology at The Agnes Ginges Center for Human Neurogenetics, Hadassah-Hebrew University Medical Center, Jerusalem, Israel; Cornea and Refractive Surgery Service, Department of Ophthalmology, Hadassah-Hebrew University Medical Center, Jerusalem, 91120 Israel

**Keywords:** Alpha linolenic acid, Nitric oxide, Conjunctival fibroblasts, Corneal epithelium

## Abstract

**Purpose:**

It is known that both human conjunctival fibroblasts (HCF) and corneal epithelial (HCE) cells contribute to the inflammatory process in the ocular surface by releasing inflammatory cytokines. In addition, nitric oxide (NO) has an important role in inflammatory responses in the ocular surface. In the present study, we aimed to characterize the capacity of these cells to release nitric oxide in response to cytokines and Lipopolysaccharide (LPS), and show that Alpha-linoleic acid (ALA) inhibits these responses.

**Methods:**

HCF, HCE cells, peripheral blood mononuclear cells (PBMCs) and co-culture of HCF and PBMC were treated with different combinations of inflammatory inducers, including interleukin)IL- (6, tumor necrosis factors (TNF)-α, interferon (IFN)- γ and IL-1β and LPS. Nitrite levels were measured in cell supernatants with and without ALA by the Griess reaction test at 24, 48 and 72 h respectively. Expression of nitric oxide synthase 2 (NOS-2) was evaluated by real-time PCR.

**Results:**

All cytokine combinations had an inducible effect on nitrite secretion in HCF, PBMC and co-cultured PBMC and HCF, but not in HCE cells. Treatment with a combination of IL-6, LPS, TNF-α, IFN- γ and IL-1β induced the highest nitrite secretion (2.91 fold, *P* < 0.01) as compared to cells incubated in medium alone. nitrite secretion was reduced by 38.9 % (*P* < 0.05) after treatment with ALA alone. Co-culturing PBMC with HCF with and without ALA treatment demonstrated similar results in nitrite level as,compared to PBMC alone. In addition, ALA significantly decreased NOS-2 expression in HCF by 48.9 % (*P* < 0. 001) after 72 h.

**Conclusions:**

The decrease in nitrite release and inhibition of NOS-2 expression indicate that ALA may have an anti-inflammatory effect both on HCF and on peripheral immune cells. This indicates that ALA may serve as a potent anti-inflammatory agent in ocular surface inflammation.

## Introduction

Polyunsaturated fatty acids (PUFAs) including alpha -linolenic acid (ALA), are the precursors of eicosanoid molecules, which are key players in inflammatory processes [1, 2]. PUFA-18:3 (n-3) derived eicosanoids have anti-inflammatory properties, while PUFA-18:2 (n-6) derived eicosanoids are considered to have pro-inflammatory characteristics [2–4].

The first evidence to examine the important that dietary intake of omega-3 PUFA plays and its effects on inflammation was derived from epidemiological observations of low incidence of myocardial infarction [5], rheumatoid arthritis and cardiovascular disease [6] in populations supplemented with n-3 PUFA. In addition, studies have confirmed that there is a relationship between oral supplementation of PUFAs and improvement in dry eye disease as well as in contact lens intolerance [7, 8]. A recent study found that topical ALA decreased the clinical signs of dry eye syndrome in a mouse model of the disease [9]. We have recently demonstrated that ALA has potent anti-inflammatory effects on human corneal epithelial (HCE) cells stimulated by Lipopolysaccharide (LPS) or polyriboinosinic:polyribocytidylic acids (poly I:C) in-vitro. Both protein and mRNA levels of several pro-inflammatory cytokines decreased following treatment with ALA [10].

Nitric oxide (NO) is a free radical which plays an important role in vasodilatation of smooth muscle, neurotransmission, and cytotoxicity [11]. In addition, inducible NO has an important role in immune and inflammatory responses [12], contributing to the acute immune response by two distinct pathways. The first pathway is direct, in which NO has a toxic effect against infectious organisms as part of the innate immune system [13]. The second is indirect, in which NO is capable of inducing or regulating the function of immune cells as part of the specific immune system [14, 15]. Previous studies related to NO’s effect in the ocular surface suggested several roles for NO, such as cell damage during infection [16, 17], pathogenesis of endotoxin-induced uveitis [18], inhibiting neovascularization [19], producing corneal edema [20], and inducing allergic reactions [21].

There are three isoforms of the Nitric oxide synthase (NOS); each has a particular function. The first two are calcium-dependent endothelial (NOS-3) and neuronal (NOS-1) enzymes, which produce low levels of NO as a cell signaling molecule in resting cells [22]. The third is the inducible calcium independent isoform (NOS-2) which is responsible for the release of NO during inflammation, and is up-regulated by a variety of extracellular stimuli such as interleukin-1β (IL-1β), tumor necrosis factor-α (TNF-α), and LPS [23, 24].

Inflammatory ocular surface diseases involve the conjunctival fibroblasts and immune cells at the ocular surface. These cells were shown to release NO at basal conditions [25], but to the best of our knowledge, these cells were not tested for their capacity to respond to immunomodulators. Therefore we have characterized the capacity of HCF and HCE cells to release nitrite at basal conditions and also after activation with immunomodulators. In addition, we investigated the anti-inflammatory effects of ALA on HCF.

## Methods

The Hadassah Medical Center Institutional Review Board (IRB) approval was obtained for this study (IRB protocol number and version: EFA-EFE-IV-01), and all of the study procedures were carried out in accordance with the IRB guidelines. This study followed the tenets of the Helsinki Declaration.

### Human Corneal Epithelial (HCE) cells and Human Conjunctival Fibroblasts (HCF)

HCE cells were obtained from human corneoscleral rim explants, taken from three different human donors, provided by the Department of Ophthalmology at the Hadassah Medical Center, using a previously described method [26]. HCE cells were cultured in supplemented hormonal epithelial medium (SHEM) [27]. HCE cells were incubated at 37 °C under 95 % humidity and 5 % CO_2_. The culture medium was replaced every other day. Cultures were kept for 10 to 14 days until a density of 90 % confluence was observed. At this time, cells were passaged and seeded onto 6-well plates at a density of 2.0 x10^5^ cells/well. Cells were observed by phase-contrast microscopy to ensure uniformity of morphology. The purity of HCE cultures was confirmed by staining for cytokeratin- 19with the indirect immunoperoxidase procedure with a monoclonal antibody to human cytokeratin-19 (Abcam, UK). Second generation cells were used in all experiments.

Human conjunctiva explant cultures were established using specimens obtained at the time of strabismus surgery. The human conjunctival explants were taken from four different human donors. The isolation and culture of cell explants were performed within 1–3 h after the strabismus surgery. HCF cells were cultured as previously described [28]. In brief, HCF cells were cultured in fibroblast medium, which contained Dulbecco’s modified Eagle medium (DMEM) with nutrient mixture F12 (Gibco), supplemented with 4 mM glutamine, 10 % fetal calf serum (FCS), 100 U of penicillin, and 100 μg of streptomycin/ml. HCF were incubated at 37 °C under 95 % humidity and 5 % CO_2_. The medium was replaced every 2–3 days. Cultures were kept for 10 to 14 days until a density of 90 % confluence was observed.

### Culture of human peripheral blood mononuclear cells (PBMCs)

Human PBMCs were isolated from buffy coats provided by the blood bank of the Hadassah Medical Center. The blood donors were four healthy males aged between 25 and 45. PBMCs were isolated as previously described [29, 30]. Briefly, whole blood was transferred via tubes covered with anticoagulant and mixed with RPMI in a 1:1 ratio. Ficoll was added to a new tube in a 1:1 ratio to the whole blood (originally before the blood/RPMI mixture), with an additional 3 mL of Ficoll. Blood/RPMI mixture was added to the Ficoll in a slow stream to maintain the gradient, and the tube was centrifuged for 30 min at 1600 rpm. The top layer (plasma) was aspirated and the buffy coat (the interface) was removed with a tube and mixed with RPMI to comprise a 50 ml total mixture. The top layer was aspirated and the cells were covered once again with new RPMI (50 ml) and centrifuged for another 10 min. The top layer was aspirated, and 1.0 ml of monocyte isolation buffer was added.

### Fatty acids

Alpha linolenic acid (ALA; 18:3 n-3) was obtained as >99 % pure sodium salt (Nuchek Prep Inc, Elysian, Minnesota, USA). ALA was dissolved in distilled water in a nitrogen chamber, filtered through a 0.2-μm-pore-size filter, divided into aliquots, and sealed under nitrogen in opaque eppendorf tubes. ALA was stored at −80 °C for no longer than 90 days before use. Fatty acids were conjugated with bovine serum albumin (BSA, Fraction V, Mercury, Israel) at a 5:1 molar ratio before treatment. In order to avoid oxidative effects, the fatty acids were defrosted once and were not reused again.

### Experimental designs

HCF and HCE cells were seeded into 6 wells plates for 72 h (density of 1.2x10^5^ cells/well) in 2.0 ml of medium. To induce inflammation, HCF and HCE cells were treated with different mixtures of pro-inflammatory cytokines and Lipopolysaccharide (1 to 100 ng/ml) combinations (Table 1). Culture medium was sampled at 24, 48 and 72 h, and nitrite accumulation was measured at each time period. HCF and HCE cells treated with combinations 1 to 5 (Table 1) were lysed at 72 h to extract RNA for RT-PCR analysis.

**Table 1:** The pro-inflammatory combinations added to cultures of HCE cells and HCF

Combination 1	LPS(10 ng/ml) + IL-6+ YNFα + IFNγ + IL-1β
Combination 2	LPS(1 ng/ml) + IL-6 + TNFα
Combination 3	LPS(10 ng/ml) + IL-6 + TNFα
Combination 4	LPS(10 ng/ml) + TNFα + IFNγ
Combination 5	LPS(100 ng/ml) + IL-6 + TNFα + IFNγ + IL-1β

The pro-inflammatory cytokines include: IFN- γ, TNF-α, IL-1β and IL-6. Each cytokines’ concentration was kept constant throughout all combinations, and was 100 ng/ml

Production of nitric oxide may promote inflammation in mononuclear cells as previously described [31]. Our preliminary results showed that PBMC released high amounts of nitrite after treatment with LPS only. Co-cultured human PBMCs (density of 2.0 x 10^5^ cells/well) with HCF in 2.0 ml of medium were treated with LPS only (1 to 1000 ng/ml LPS). In addition, co-cultured PBMC with HCF were stimulated by LPS at similar conditions to those of PBMC and HCF alone. Culture medium was sampled at 24, 48 and 72 h. Nitrite accumulation was measured at each time period.

HCF, HCE cells, and human PBMCs were pre-incubated for two hours with 200 μM ALA before treatment with the inflammatory stimulus [32].

### Griess reaction

NO is unstable in culture medium. Nitrite accumulation, an indicator of NO synthesis, was measured in the culture medium by Griess reaction at 24, 48 and 72 h [33]. Briefly, 80 μL of cell culture medium was mixed with 20 μL of Griess reagent [equal volumes of 1 % (w/v) sulfanilamide in 5 % (v/v) phosphoric acid and 0.1 % (w/v) naphthylethylenediamine-HCl] and incubated at room temperature for 10 min. The absorbance at 550 nm was then measured using a microplate reader. Fresh culture medium was used as a blank in all experiments. The amount of nitrite in the test samples was calculated from a sodium nitrite standard curve.

### RNA isolation

Following treatment, total RNA was extracted from the HCF and HCE cell samples with RNAqueous Kit (Ambion, USA) following the manufacturer’s instructions. Quantification of total RNA was performed in a NanoDrop spectrophotometer (ND-1000, USA). RNAs were stored at −80 °C until further utilization.

### cDNA synthesis

cDNA was synthesized from purified and concentrated 0.5 μg RNA from each sample using a High Capacity cDNA Reverse Transcription Kit (Applied Biosystems, ABI, USA). A 20 μl total reaction volume was made with 10 μl RNA, 2 μl 10X RT buffer, 0.8 μl dNTP Mix (100 mM), 2.0 μl 10X RT random hexamer primers, 1.0 μl MultiScribe™ reverse transcriptase, 1 μl RNase inhibitor and 3.2 μl nuclease-free water. Synthesis was carried out in an ABI 7900 Thermo cycler (ABI, USA) and RT-PCR reaction conditions were comprised of: 25 °C for 10 min, 37 °C for 120 min, and 85 °C for 5 mins. cDNA samples were stored at −20 °C until use.

### Real-time polymerase chain reaction

Real-time polymerase chain reaction (Real-time PCR) was performed using TaqMan ® Gene Expression Assays (ABI, USA) in the ABI Prism 7900HT Sequence Detection System (ABI, USA) as previously described [34, 35]. Negative controls were included to evaluate DNA contamination of isolated RNA and reagents.

Real-time PCR assays for iNOS probe (ABI, USA) were performed. An amount of 1 μL cDNA was loaded in each reaction, to a total volume of 20 μl of reaction mixture, and assays were performed in triplicates.

The fold change in gene expression was normalized to the expression of the endogenous gene hypoxanthine phosphoribosyltransferase 1 (HPRT1; ABI, USA). Quantitative analysis was performed using the comparative (ΔΔC_T_) method [34, 35] The results were analyzed by DataAssist™ SoftwareV 2.0 (ABI, USA).

### Statistical analysis

All tests were carried out on three independent cell cultures, and performed in triplicate for each of the treatments. In the experiments of the effect of ALA on nitrite secretion in HCF and effect of ALA on NOS-2 mRNA expression tests, we carried out the tests on four independent cell cultures, and performed triplicates for each of the treatments.

Statistical analysis and multiple comparisons were performed by one-way ANOVA using the InStat software version 3.0 (InStat software Inc., Graphpad, San Diego, CA). The differences in mean values among treatment groups were determined by ANOVA adjusted for multiple comparison by the Tukey test, ANOVA adjusted for multiple comparison by the Student-Newman-Keuls test, and ANOVA with multiple comparison by the Bonferroni *t*-test.

## Results

### Nitrite levels induction in HCF and HCE cells

**Fig. 1:**
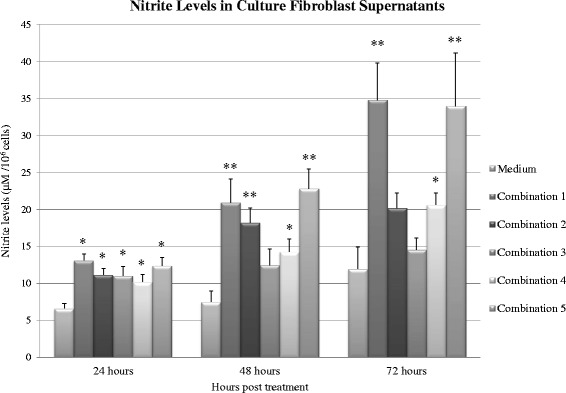
The levels of nitrite accumulation in the medium of HCF induced by LPS at different concentrations with different combinations of cytokines (Table 1) as measured by the Griess reaction. Measurements were performed at 24 h, 48 h and 72 h after applying the inflammatory combinations. Each bar represents the mean and SD of media nitrite concentrations of triplicate cultures after 24, 48 and 72 h (n = 3). The asterisk (*P* < 0.05) and double asterisk (*P* < 0.01) represent statistical significance for experiments vs medium alone

In order to induce nitrite synthesis in HCF and HCE cells, we used 5 different combinations of cytokines and LPS as depicted in Table 1. Primary cultures of HCF from three individual corneas were incubated with the 5 different combinations of stimulators (n = 3). Of these combinations, combinations #1 and #5 were found to be the optimal combinations that caused the highest nitrite secretion after 24–72 h (Fig. 1).

We observed 20.93 ± 3.1 and 22.82 ± 2.65 fold higher levels of nitrite in combination #1 and #5 respectively (*P* < 0.01) after 48 h of stimulation. After 72 h of stimulation we observed 34.81 ± 5.0 and 33.9 ± 7.17 fold higher levels of nitrite in combination #1 and #5 respectively (*P* < 0.01). Therefore, we chose to use treatment with combination #1 for 72 h in HCF for further experiments, as it produced the highest levels of nitrite.

The HCE cells that were treated with the same combinations of cytokines and LPS showed very low amounts of nitrite accumulation after 24 to 72 h.

### Effect of ALA on Nitrite secretion in HCF and PBMCs

We wished to determine if there was a beneficial effect if ALA was added to cells in an inflammatory environment. Therefore, we incubated HCF with ALA for 2 h and then exposed the cells to 10 ng/ml LPS and cytokines as was described in combination # 1. Nitrites were evaluated in supernatants after 72 h (n = 3).

**Fig. 2:**
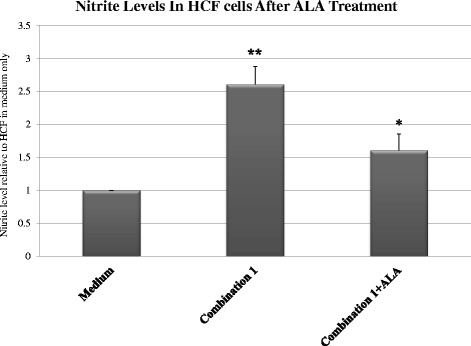
Nitrite accumulation in stimulated cultured HCF cells with and without incubation with ALA. HCF cells incubated with ALA for 2 h and then exposed to 10 ng/ml LPS with cytokines, as described in combination 1. Nitrites were evaluated in supernatants after 72 h (n = 3). The asterisk (*P* < 0.01) represents statistical significance (*P* < 0.01) for stimulated HCF cells stimulated after treatment with ALA vs stimulated HCF cells without ALA. The double asterisk represents statistical significance (*P* < 0.01) for stimulated HCF cells vs HCF cells in medium alone. ALA - alpha-linoleic acid; HCF - human conjunctival fibroblasts

We compared the nitrite accumulation in supernatants of HCF cultures incubated with or without ALA before combination #1 treatment. Induced HCF showed 2.6 fold higher levels of nitrite in excess of control levels (*P* < 0.05) (controls comprised normal HCF receiving no pro-inflammatory combinations, or ALA treatment), while in ALA-treated cultures, we observed 1.6 fold higher levels (*P* > 0.05) of nitrite compared to controls. There was a 38.9 % decrease (*P* < 0.05, Student-Newman-Keuls test) in nitrite secretion in inflammatory-induced HCF cultures treated with ALA as compared to HCF not treated with ALA (Fig. 2).

Our preliminary results showed that PBMC released high amounts of nitrite after treatment with LPS alone. We have shown that in order to cause a significant production of nitrite in HCF we need the combination of cytokines.

Cultured human PBMCs were stimulated by LPS (LPS B4) at a dose of 1000 ng/ml (n = 4). After 72 h, culture medium was extracted, and nitrite levels were measured. LPS concentration and incubation period were chosen dependent on maximum nitrite secretion (data not shown).

**Fig. 3:**
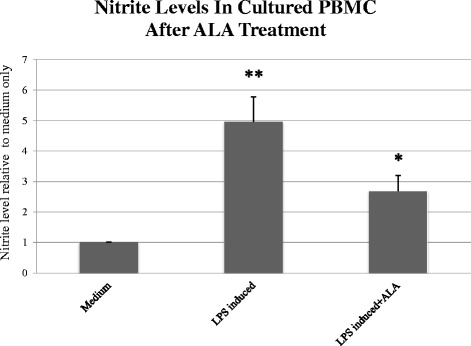
Nitrite accumulation in LPS-induced peripheral blood mononuclear cells (PBMCs) with and without incubation with ALA. Cultured human PBMCs were induced by LPS (LPS B4) at a dose of 1000 ng/ml. After 72 h, the culture medium was extracted and nitrite levels were measured (n = 4). The asterisk (*P* < 0.01) represents statistical significance for PBMCs stimulated using LPS after treatment with ALA vs PBMCs stimulated using LPS alone. Double asterisk (*P* < 0.001) represents statistical significance for PBMCs stimulated with LPS vs PBMC in medium alone. LPS - Lipopolysaccharide, ALA - alpha-linoleic acid; PBMC - peripheral blood mononuclear cell

After incubating cultured human PBMC with and without ALA, the cells were induced once again by LPS at a dose of 100 ng/ml for a period of 72 h (n = 4). In PBMCs stimulated by LPS after ALA treatment, nitrite levels were reduced by 2.67 ± 0.519 folds compared to 4.95 ± 0.81 fold in PBMCs incubated in medium alone (*P* > 0.05; Fig. 3).

**Fig. 4:**
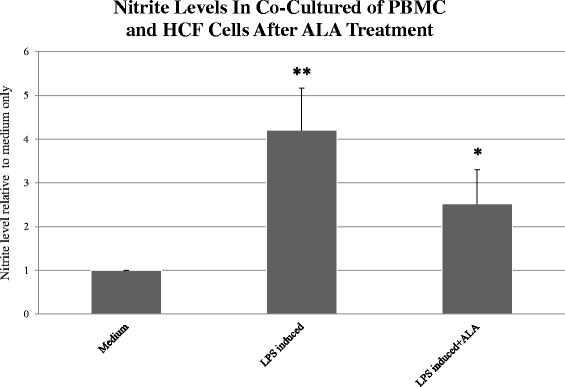
Nitrite accumulation in LPS-induced co-culture of peripheral blood mononuclear cells (PBMCs) and HCF cells with and without treatment of ALA. Cultured human PBMC and HCF cells were induced by LPS (LPS B4) at a dose of 1000 ng/ml. After 72 h, the culture medium was extracted and nitrite levels were measured (n = 4). The asterisk (*P* < 0.05) represents statistical significance for PBMCs stimulated using LPS after treatment with ALA vs PBMC and HCF cells stimulated using LPS alone. Double asterisk (*P* < 0.001) represents statistical significance for PBMC stimulated with LPS vs PBMC in medium alone. LPS - Lipopolysaccharide, ALA - alpha-linoleic acid; PBMC - peripheral blood mononuclear cells; HCF - human conjunctival fibroblasts

Co-culturing PBMC with HCF that was induced by LPS demonstrated similar results in nitrite level compared to PBMC alone. In co-cultured cells induced by LPS, nitrite accumulation was 4.2 ± 0.96 folds higher compared to co-cultured cells incubated in medium alone. Following ALA treatment, nitrite levels were reduced to 2.51 ± 0.78 folds compared to cells in medium alone (*P* > 0.05; Fig. 4).

### The effect of ALA on NOS-2 mRNA expression

**Fig. 5:**
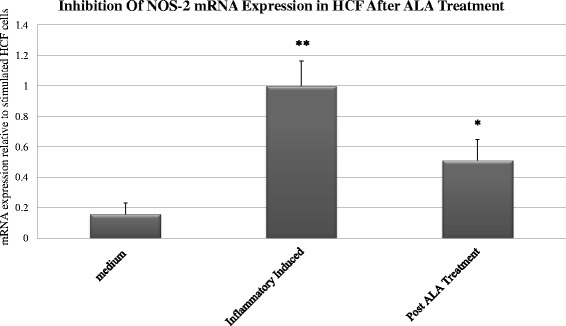
Inhibition of NOS-2 expression in inflammatory-induced HCF cells by Alpha-linoleic acid (ALA). Cultures of HCF cells were stimulated using combination 1 (Table 1) for 72 h treatment and NOS-2 mRNA levels were measured using Real-time PCR (n = 4). Each bar represents the mean SD of NOS-2 expression in quadruplicate cell cultures. The asterisk (*P* < 0.01) represents statistical significance for HCF cells stimulated using combination 1 after ALA treatment vs HCF cells stimulated using combination 1. Double asterisk (*P* < 0.001) represents statistical significance for HCF cells stimulated with combination 1 vs HCF cells in medium alone. LPS - Lipopolysaccharide, ALA - alpha-linoleic acid; HCF – human conjunctival fibroblasts; NOS-2 - nitric oxide synthase 2

Along with the investigation into the release of nitrite, we evaluated the expression of NOS-2 in cells induced by combination 1 treatment for 72 h (LPS B4 10 ng/ml + IL6 100 ng/ml + TNFα 100 ng/ml + IFN γ 100 ng/cc + IL1β 100 ng/cc), which induced the highest nitrite secretion, as compared to the other four combinations. Real-time PCR confirmed that NOS-2 mRNA was induced in cultures treated with LPS and pro-inflammatory cytokines. Measurement of NOS-2 mRNA was made in cultured HCF that were induced by combination # 1 with (n = 4) and without (n = 4) incubation with ALA. Cultured HCF induced by combination # 1 showed an increase in NOS-2 mRNA expression compared to cells incubated in medium alone (*P* < 0.001), and was normalized to 1. Treating inflammatory-induced HCF cultures with ALA induced a 48.9 % decrease in NOS-2 mRNA expression compared to inflammatory-induced HCF not treated with ALA (*P* < 0.01) as measured by real-time PCR (Fig. 5).

## Discussion

This study demonstrates that a combination of inflammatory cytokines has an recognizable effect on nitrite secretion in HCF up to 48 h post-induction. Induction with a combination of IL-6, LPS, TNF-α, IFN- γ and IL-1β produced the highest nitrite secretion up to 72 h in vitro. Our data also demonstrates the production of NOS-2 mRNA expression in HCF induced by the same combination, indicating that NOS-2 is responsible for the induction of NO. Our preliminary results showed that PBMC released high amounts of nitrite after treatment with LPS, unlike HCF. In order to cause a significant production of nitrite in HCF, the cells need to be stimulated with a combination of cytokines and LPS. Nitrite levels were found to be significantly higher in LPS-induced PBMCs as compared to induced cultured HCF. Treating inflammatory-induced HCF and PBMC cultures with ALA showed a significant decrease in nitrite levels, and this impact was even larger on PBMC cultures.

In the first stage of our work, we induced an inflammatory reaction in HCF and HCE cells by using a combination of cytokines and LPS. We did not use single cytokines or LPS alone, due to earlier findings on animal models showing that the combination of cytokines and LPS up-regulate NOS-2 expression and cause an accumulation of nitrite in significantly higher levels as compared to treatment with a single cytokine or LPS alone [36, 37]. In HCF cultures, our findings demonstrate that all combinations of treatment cause a significant increase in nitrite levels as soon as 24 h post-induction, as noticed in earlier studies [37]. In agreement with previous studies [37], we did not find a significant elevation in nitrite secretion by HCE cells when an inflammatory reaction was induced.

We showed that IL-1β has an additional inductive effect when added to TNF-α, IFN- γ and LPS in both 48 h and 72 h stimulation. This is opposed to a study by Dighiero et al. [36] that showed no significant difference in nitrite secretion in basal cell epithelioma and keratocytes when adding IL-1β to, TNF-α, IFN- γ LPS.

In our study, the combination of IL-6, LPS, TNF-α, IFN- γ and IL-1β that induced the highest nitrite secretion, elicited 2.91-fold higher levels of nitrite after 72 h, compared to cells incubated in medium alone. O’Brian et al. [37] showed a similar effect on rabbit corneal stromal cells using the same combination, without IL-6, to elicit 5-fold higher levels of NO, compared to cells incubated in medium alone. These results can be possible due to the fact that it is known that different pro-inflammatory cytokines, as well as LPS, have specific effects on different cell types, and are species dependent [38].

Kim et al. [25] induced ocular inflammation in rabbits in-vivo and showed that stromal fibroblasts and inflammatory cells are the main source of NO in ocular inflammation. In addition, Kim et al. examined the levels of NO in tears. According to the study, if the concentration ratio of NO is 1.5-2.5 fold higher from the normal NO functional level (the normal functional NO level was defined as 1.0 in that study), NO may play a defensive role. However, if the concentration ratio of NO is 3–10 folds higher, NO may induce tissue damage. In our study, HCF induce a relative low level of nitrite compared to PBMCs. At these levels, NO may have a role in the normal healing process, or as a defense mechanism inducing cell survival. On the other hand, PBMCs that induce high levels of nitrite compared to HCF may take part in pathologic inflammation causing tissue damage, oxidative stress, and DNA damage [25]. We may assume, therefore that HCF, which are regularly exposed to potential allergens and foreign substances, demonstrate a constant protective effect on the ocular surface, promoting quiescence and maintaining normal homeostasis by secreting NO at low levels. Our findings regarding the protective role of the conjunctiva match earlier findings showing a nearly 100-fold increase in Interleukin 10 (IL-10) expression in ocular inflammation [9]. IL-10 inhibits both innate and T-cell–mediated immunity.

HCF secreted low levels of nitrite relative to PBMC. In addition, co-culturing PBMCs with HCF induced by LPS demonstrated similar results in nitrite levels compared to PBMC alone. This finding indicates that PBMCs could be the main source of NO release in inflammatory events.

The ability of cells and tissues to regulate the production and accumulation of NO in large amounts (accumulations of 10 mM or greater), is by NOS-2, a 130 kDa protein which is primarily regulated at the transcriptional level. Our data documents the production of NOS-2 mRNA expression, and nitrite accumulation in cytokine/LPS-treated HCF cultured for 4 passages. The elevated NOS-2 expression coincided in time with the accumulation of nitrites. The ability to induce NOS-2 in HCF required the presence of pro-inflammatory cytokines and LPS. When comparing the nitrite accumulation to the NOS-2 mRNA accumulation in HCF treated with the same cytokine combination, there is a mismatch between the significantly elevated NOS-2 expression compared to the relatively slight rise in nitrite levels in medium culture. Vodovotz et al. [39] noted that mouse peritoneal macrophages incubated with IFN- γ and LPS, though initially producing NOS-2 protein and NO, eventually, have a post-translational and non-degradative inactivation of the enzyme. These cultured cells with inactivated enzyme are somewhat comparable with what was observed in HCF, expression of high levels of NOS-2 mRNA, compared to low NO production. This group also noted that the inactivation of NOS-2 required the presence of LPS.

The role of NO in the pathogenesis of inflammatory eye diseases is not well understood. In our work, we indicate that PBMCs released a high concentration of nitrite relative to HCF. Our findings may corroborate the results by Kim et al. that relatively low NO concentration (released from HCF) during inflammation is probably part of normal wound healing, and serves as a defense mechanism inducing cell survival [25].

The clinical data from studies on the systemic effects of orally-administered n-3 PUFA, with the in-vivo murine study, demonstrate significant systemic and local anti-inflammatory effects of n-3 PUFA [2–4].

Our results show that ALA, an n-3 PUFA, has a significant inhibitory effect on the nitrite level secretion by HCF, PBMC and co-culture of HCF and PBMC. These findings can be explained by considering two earlier studies: One by Xie et al. [40] which demonstrated that NF-kappa B primarily regulates NO secretion through iNOS gene expression [40], and the second was our previous study, which demonstrated that ALA decreased NF-κB activity through Inhibitory factor-κBα (I-κBα) mRNA expression in HCE cells [10].

In conclusion, in our study we have found evidence that demonstrated that both nitrite levels and the expression of the mRNA of NOS decreased after ALA treatment. The decrease in nitrite release and inhibition of NOS-2 expression indicates that ALA may have an anti-inflammatory effect both on HCF and on peripheral immune cells. This knowledge brings us one-step forward towards treatment of inflammation in ocular diseases and decrease of the pathological mediators using anti-inflammatory compounds such as ALA.
